# Validation of Claims Algorithms for Progression to Metastatic Cancer in Patients with Breast, Non-small Cell Lung, and Colorectal Cancer

**DOI:** 10.3389/fonc.2016.00018

**Published:** 2016-02-01

**Authors:** Beth L. Nordstrom, Jason C. Simeone, Karen G. Malley, Kathy H. Fraeman, Zandra Klippel, Mark Durst, John H. Page, Hairong Xu

**Affiliations:** ^1^Evidera, Lexington, MA, USA; ^2^Amgen, Inc., Thousand Oaks, CA, USA

**Keywords:** cancer progression, metastatic cancer, claims algorithm, oncology, random forests

## Abstract

**Background:**

Validated algorithms for identifying progression to metastatic cancer could permit the use of administrative claims databases for research in this area.

**Objective:**

To identify simple algorithms that could accurately detect cancer progression to metastatic breast, non-small cell lung, and colorectal cancer (CRC) using medical and pharmacy claims data.

**Methods:**

Adults with stage I–III breast, non-small cell lung cancer (NSCLC), or CRC in the Geisinger Health System from 2004 to 2011 were selected. Evidence of progression was extracted *via* manual chart review as the reference standard. In addition to secondary malignancy diagnosis (ICD-9 code for metastases), diagnoses, procedures, and treatments were selected with clinician input as indicators of cancer progression. Random forests models provided variable importance scores. In addition to codes for secondary malignancy, several more complex algorithms were constructed and performance measures calculated.

**Results:**

Among those with breast cancer [17/502 (3.4%) progressed], the performance of a secondary malignancy code was suboptimal [sensitivity: 64.7%; specificity: 86.0%; positive predictive value (PPV): 13.9; negative predictive value (NPV): 98.6%]; requiring malignancy at another site or initiation of immunotherapy increased PPV and specificity but decreased sensitivity. For NSCLC [61/236 (25.8%) progressed], codes for secondary malignancy alone (PPV: 47.4%; NPV: 84.8%; sensitivity: 60.7%; specificity: 76.6%) performed similarly or better than more complex algorithms. For CRC [33/276 (12.0%) progressed], secondary malignancy codes had good specificity (92.7%) and NPV (92.3%) but low sensitivity (42.4%) and PPV (43.8%); an algorithm with change in chemotherapy increased sensitivity but decreased other metrics.

**Conclusion:**

Selected algorithms performed similarly to the presence of a secondary tumor diagnosis code, with low sensitivity/PPV and higher specificity/NPV. Accurate identification of cancer progression likely requires verification through chart review.

## Introduction

Cancer progression is an important outcome in oncology research. Administrative claims databases, which are frequently used for retrospective studies of treatment patterns, safety, and effectiveness of cancer therapies, have no data that can directly indicate cancer progression. Metastatic cancer is to some extent identifiable through secondary tumor International Classification of Diseases, Ninth Revision (ICD-9) codes, but the accuracy and completeness of these codes in claims data are known to be poor (1).

Numerous algorithms have been published that identify incident breast, lung, and colorectal cancers (CRCs) (2–10). However, identifying metastatic cancer has proven more challenging than identifying incident cancers. One study using linked surveillance, epidemiology, and end results (SEER) and Medicare data found good positive predictive value (PPV) for identifying incident breast cancer overall but demonstrated fairly low sensitivity (51%) in the subset of patients with stage IV cancer (5); similar findings of good specificity but low sensitivity were reported in a recent claims database study identifying metastatic breast, lung, and CRC (11). Another study used logistic regression modeling with the SEER–Medicare data to predict stage IV breast cancer; this model achieved reasonable sensitivity (81%) and specificity (89%) but had poor PPV (24%) (8). The identification of progression to metastatic cancer is even more complex than identifying metastatic cancer, as data are required on both the change from the initial stage at diagnosis to metastasis and the timing of this change.

Chubak et al. developed algorithms to identify second breast cancer events (recurrence or second primary breast cancer) among women enrolled in an integrated health-care system from 1993 to 2006 (12). One algorithm had high specificity (99%) but lower sensitivity (89%) and PPV (90%); a different algorithm achieved a higher sensitivity (96%), but lower specificity (95%) and PPV (74%). A study of patients in the Kaiser Permanente Southern California Health System used a SAS-based coding, extraction, and nomenclature tool to identify primary and recurrent breast and prostate cancers from pathology reports (13). The sensitivity, specificity, and PPV of this tool exceeded 94% for both cancer types. McClish and colleagues merged Medicare inpatient and Part B data with cancer registry data and used a logistic regression model to identify predictors of recurrent cancer (14). In this study, predictors of cancer recurrence included regional or distant stage disease, diagnosis of secondary malignancy, and an inpatient diagnosis of primary cancer in a secondary position.

A means of identifying progression to metastatic cancer in claims data would make it possible to conduct claims based oncology research taking this critical outcome into account. Using information from patient charts linked to medical and pharmacy claims data, the present study sought simple algorithms that could accurately identify cancer progression to metastatic disease in claims data alone. Separate algorithms were developed for three of the most common tumor types in the database.

## Materials and Methods

### Data Sources

The Geisinger Health System includes a single-state 700+ multi-specialty physician group practice, 3 hospitals, 40 ambulatory clinics, a clinical reference laboratory, a non-exclusive health plan, and 3 dedicated research centers. The electronic health record (EHR) data include inpatient and outpatient care, as well as laboratory results, vital signs, and lifestyle data, including smoking status. A cancer registry database includes clinical details, such as tumor size, grade, stage, and antineoplastic treatments. The cancer registry data, with supplemental manual review of inpatient and outpatient charts to confirm stage at diagnosis and occurrence or non-occurrence of cancer progression, provided the reference standard data for this analysis.

Claims data were obtained from the Geisinger Health Plan, which provides coverage for a subset of patients seen in the Geisinger Health System. The claims database covers all covered services from all sites of care and contains the usual data found in a claims database (e.g., enrollment dates, diagnosis and procedure codes, and pharmacy-dispensed medications).

Because the study involved only de-identified, retrospective data, it was exempt from IRB approval, and informed consent of patients was not required.

### Patient Selection

Patients were required to meet all of the following inclusion criteria:

- An initial diagnosis in the cancer registry of one of the following primary cancer types between January 1, 2004 and December 31, 2011, with stage at diagnosis indicated as I, II, or III:
  ○ Breast [females only; International Classification of Dis­eases for Oncology, Third Edition (ICD-O-3) code C50.x]
  ○ Non-small cell lung cancer (NSCLC; C34.x, C33.9)
  ○ Colorectal (C18.x, C19.9, C20.9, C21.0–C21.2, C21.8)
- Adults (≥18 years of age at date of initial cancer diagnosis)
- Enrolled in the Geisinger Health Plan at the time of initial diagnosis with cancer.

The date of initial cancer diagnosis was designated as the index date. Patients with a second primary tumor type on or before the index date, those whose continuous enrollment ended fewer than 90 days after the index date, and those diagnosed with metastatic cancer before or within 60 days after the index date (i.e., during the initial workup for cancer) were excluded.

### Outcome Definition

The study outcome was cancer progression to distant metastasis, defined as a cancer that has spread from the primary site to distant tissues or organs or to distant lymph nodes (15). To ascertain the outcome, medical record review was conducted for all patients enrolled in the study cohort. Inpatient and outpatient charts were reviewed, with particular emphasis on radiology, surgery, and pathology reports. The following data elements were extracted:

- Primary tumor site
- Diagnosis of metastatic (stage IV) cancer
- Date of diagnosis of stage IV cancer
- Locations of metastases
- Diagnostic methods used to diagnose metastases
- Any applicable supporting notes on metastasis diagnosis.

Research nurses who were trained in oncology extracted the clinical information using a standardized extraction form. De-identified data from completed abstraction forms were entered into the research database using a unique ID number for each patient.

### Variable Definitions

Patients were followed through the claims data from the index date to the earliest of death, disenrollment from the health plan, or end of the study period to create variables considered for the claims algorithm. An initial algorithm was constructed with guidance from an oncologist. This initial algorithm specified the presence of at least one radiological or pathological claim ≥60 days after the index date followed by at least one element derived from procedures, diagnoses, and medications that could indicate tumor progression to distant metastatic cancer. Indicator variables for drugs, diagnoses, and procedures were created with separate sets of variables for breast, lung, and CRC.

The variables included in the initial algorithm were events that occurred within 30 days after a radiology or pathology claim, where the radiology/pathology claim was required to occur at least 60 days after the index date; sensitivity analyses extended this time window to 90, 120, 180, and 365 days. The following variables were created:

- Diagnosis of secondary malignancy
- Diagnosis of a primary malignancy at a different site from the original tumor (malignancy of the contralateral breast or lung did not qualify as a different site)
- Another radiological procedure at a different site than the primary tumor
- Biopsy of site other than the primary tumor site
- Surgery for tumor excision at a new site or second surgery at the same site
- Use of pain medication (opioids)
- Use of medications for bone disease (e.g., bisphosphonates)
- Use of systemic corticosteroids
- Ambulatory aids (e.g., wheelchair)
- Airway management, for lung cancer only
- Feeding (parenteral nutrition), for lung cancer only
- Radiation therapy (with or without prior radiation therapy)
- Initiation of radiation therapy for patients with no prior history of this treatment
- Initiation of hormonal therapy, for breast cancer only
- Initiation of immunotherapy, for breast cancer only
- Change in chemotherapy from prior regimen
- Initiation of metastatic cancer-specific therapy
- Change from a multidrug regimen to a single antineoplastic agent
- Various diagnoses related to pain, confusion, seizures, or fractures, or (for breast cancer only) a lump/swelling or shortness of breath.

### Statistical Analysis

A separate algorithm consisting of a radiology or pathology claim followed by one or more of the variables listed previously was developed for each tumor type, with the cancer progression data obtained *via* chart review serving as the reference standard. The algorithm was developed through a hybrid clinical and empirical approach. Clinical insight drove each step of the process, with statistical methods used to test and refine the algorithm.

We first used random forests (RF) to evaluate the relative importance of variables in order to reduce a large set of potential predictor variables to a more parsimonious subset (16, 17). RF is a machine learning technique that grows a forest of decision trees, aggregates them, and yields a predicted status of metastatic or non-metastatic cancer for each patient. RF incorporates randomness by sampling patients and variables to build the decision trees. The prediction accuracy of a tree is tested by classifying patients who were not used to build a tree and computing the misclassification rate. RF uses a permutation strategy to rank the importance of a variable to the prediction by measuring the decrease in prediction accuracy when the values of that variable are randomly permuted (18). The greater the loss of accuracy (i.e., more mismatches of patients as progressed or not progressed), the more important the variable is to the prediction. Because there are many more non-metastatic than metastatic patients, we pre-balanced the group sizes for each forest by randomly sampling from the larger group. Then, we ran 100 forests, using a new random selection of the non-metastatic patients each time, and averaged the results. Each forest had 1000 trees.

Using the variable importance results for each tumor type and each time window for the minimum time to the qualifying radiology/pathology claims, and applying clinical judgment to the combinations of variables with high importance, we selected a small number of predictor sets for each tumor type. One predictor set consisted of a single variable, which was established *a priori* as the ICD-9 code for secondary malignancy (i.e., metastasis); others included just two or three variables.

The single-variable model was evaluated using a simple 2 × 2 table and associated performance measures, including sensitivity, specificity, PPV, and negative predictive value (NPV) (19). Two- or three-variable models were each used in a preliminary RF run to identify the forest error rates based on this small set of variables, and to look for highly predictive trees. To identify such trees, the preliminary forest was saved, and the 1000 saved decision trees from the forest were used in a second RF run, in which the trees were the predictor “variables.” This is called a synthetic forest (20); it is a way of evaluating the 1000 branching algorithms from the preliminary forest. The importance rankings of the trees enabled the identification of branching algorithms that performed consistently well. Since the saved forest only used a random sample of the larger (non-metastatic) group, this procedure was run multiple times. Forests with error rates widely different from the preliminary run were not considered. Of all algorithms tested, those that ranked consistently highly (i.e., those with the lowest error rates) were selected for further review.

The selected algorithms were applied to the full data set, and performance measures were calculated for each of the resulting 2 × 2 tables. Data management and initial analyses were performed in SAS version 9.4 (21). The RF procedure was conducted in R version 3.3.1 (22) with the randomForests package version 4.6-10 (23).

## Results and Discussion

A total of 1357 patients were diagnosed with breast, NSCLC, or CRC from January 1, 2004 to December 31, 2011. After applying all inclusion and exclusion criteria, 1017 patients were eligible for the analysis, of whom 502 were diagnosed with breast cancer, 236 with NSCLC, and 279 with CRC.

The median and interquartile range (IQR) of age of patients were 62 (51–72) years for breast cancer, 72 (65–78) years for NSCLC, and 71 (61–78) for CRC (Table 1). While all included patients with breast cancer were women, men composed of the majority of patients diagnosed with NSCLC (56.8%) and CRC (52.0%). Over 98% of patients with all tumor types were non-Hispanic whites. NSCLC patients were more likely have a record indicating current or former smoking (76.3%) when compared to those with breast (31.5%) or CRC (40.9%), although nearly 20% of patients were missing data on smoking status. Median (IQR) follow-up enrollment in the Geisinger Health Plan, in months after the index date, was 35.7 (20.0–59.9) for breast cancer, 17.6 (8.6–33.1) for lung cancer, and 30.7 (16.5–53.4) for CRC.

**Table 1:** **Patient characteristics**.

Patient characteristic at index date	Breast cancer (*N* = 502)	Non-small cell lung cancer (*N* = 236)	Colorectal cancer (*N* = 279)
**Age at index (years)**			
Mean (SD)	61.4 (13.3)	70.7 (9.3)	68.5 (12.5)
Median (range)	62 (30–89)	72 (43–89)	71 (23–89)
**Gender**			
Male	0 (0.0%)	134 (56.8%)	145 (52.0%)
Female	502 (100.0%)	102 (43.2%)	134 (48.0%)
**Ethnicity/race**			
White, not Hispanic	494 (98.4%)	234 (99.2%)	278 (99.6%)
Other	7 (1.4%)	2 (0.8%)	1 (0.4%)
Missing	1 (0.2%)	0 (0.0%)	0 (0.0%)
**Smoking status at index**			
Current/former	158 (31.5%)	180 (76.3%)	114 (40.9%)
Never	256 (51.0%)	14 (5.9%)	103 (36.9%)
Missing	88 (17.5%)	42 (17.8%)	62 (22.2%)

*All cells are *n* (%) unless otherwise specified*.

### Progression Algorithms

#### Breast Cancer

Only 17 of the breast cancer patients (3.4%) progressed to metastatic cancer. Nearly all patients, including all of the patients who progressed, had a radiology or pathology claim ≥60 days after their initial cancer diagnosis (Table 2). Within 30 days following a qualifying radiology/pathology claim, a diagnosis of secondary malignancy, diagnosis of a second primary tumor, and initiation of immunotherapy appeared to discriminate most clearly between patients with and without progression to stage IV disease. The RF results supported this, with variable importance values that were highest for these three indicators.

**Table 2:** **Variables considered for cancer progression algorithm**.

Presence of characteristic for progression algorithm	Breast cancer (*N* = 502)		Non-small cell lung cancer (*N* = 236)		Colorectal cancer (*N* = 279)	
	Did not progress to Stage IV (*N* = 485)	Progressed to Stage IV (*N* = 17)	Did not progress to Stage IV (*N* = 175)	Progressed to Stage IV (*N* = 61)	Did not progress to Stage IV (*N* = 246)	Progressed to Stage IV (*N* = 33)
**Initial element, required within 30 days before each of the following elements**						
Radiological or pathological claim ≥60 days after index day	481 (99.2%)	17 (100.0%)	171 (97.7%)	61 (100.0%)	236 (95.9%)	33 (100.0%)
**Additional elements following radiology/pathology claim**						
Secondary malignancy	68 (14.0%)	16 (94.1%)	41 (23.4%)	53 (86.9%)	18 (7.3%)	28 (84.8%)
Malignancy at different site	55 (11.3%)	12 (70.6%)	35 (20.0%)	36 (59.0%)	35 (14.2%)	19 (57.6%)
Another radiological procedure from non-primary tumor site	387 (79.8%)	17 (100.0%)	157 (89.7%)	60 (98.4%)	175 (71.1%)	32 (97.0%)
Biopsy claim from non-primary tumor site	77 (15.9%)	10 (58.8%)	27 (15.4%)	15 (24.6%)	29 (11.8%)	19 (57.6%)
More invasive surgery	26 (5.4%)	1 (5.9%)	2 (1.1%)	0 (0.0%)	30 (12.2%)	4 (12.1%)
Surgery on non-primary site	67 (13.8%)	3 (17.6%)	11 (6.3%)	6 (9.8%)	8 (3.3%)	7 (21.2%)
Pain medication	250 (51.5%)	10 (58.8%)	88 (50.3%)	47 (77.0%)	113 (45.9%)	21 (63.6%)
Medication for bone disease and bone pain	60 (12.4%)	2 (11.8%)	0 (0.0%)	5 (8.2%)	2 (0.8%)	0 (0.0%)
Corticosteroids	284 (58.6%)	15 (88.2%)	110 (62.9%)	51 (83.6%)	89 (36.2%)	22 (66.7%)
Wheelchair/aids for walking assistance	39 (8.0%)	4 (23.5%)	24 (13.7%)	16 (26.2%)	23 (9.3%)	5 (15.2%)
Radiation therapy (any)	358 (73.8%)	14 (82.4%)	74 (42.3%)	43 (70.5%)	42 (17.1%)	19 (57.6%)
Airway management	N/A	N/A	3 (1.7%)	5 (8.2%)	N/A	N/A
Feeding	N/A	N/A	1 (0.6%)	0 (0.0%)	N/A	N/A
Initiation of radiation therapy	138 (28.5%)	9 (52.9%)	26 (14.9%)	26 (42.6%)	12 (4.9%)	12 (36.4%)
Initiation of hormonal therapy	189 (39.0%)	4 (23.5%)	N/A	N/A	N/A	N/A
Initiation of immunotherapy	28 (5.8%)	8 (47.1%)	N/A	N/A	N/A	N/A
Change in chemotherapy	154 (31.8%)	12 (70.6%)	61 (34.9%)	38 (62.3%)	56 (22.8%)	29 (87.9%)
Initiation of metastatic-specific therapy	93 (19.2%)	8 (47.1%)	14 (8.0%)	16 (26.2%)	8 (3.3%)	20 (60.6%)
Change from multidrug regimen to single antineoplastic agent	36 (7.4%)	6 (35.3%)	5 (2.9%)	8 (13.1%)	N/A	N/A
Bone pain	54 (11.1%)	2 (11.8%)	12 (6.9%)	12 (19.7%)	10 (4.1%)	4 (12.1%)
Specific (non-bone) pain	128 (26.4%)	9 (52.9%)	N/A	N/A	N/A	N/A
Other pain	5 (1.0%)	1 (5.9%)	N/A	N/A	N/A	N/A
Confusion	0 (0.0%)	0 (0.0%)	1 (0.6%)	0 (0.0%)	0 (0.0%)	0 (0.0%)
Seizures	0 (0.0%)	0 (0.0%)	2 (1.1%)	0 (0.0%)	0 (0.0%)	0 (0.0%)
Fractures	41 (8.5%)	1 (5.9%)	13 (7.4%)	7 (11.5%)	31 (12.6%)	1 (3.0%)
Lump/mass/swelling	21 (4.3%)	2 (11.8%)	N/A	N/A	N/A	N/A
Shortness of breath	64 (13.2%)	5 (29.4%)	N/A	N/A	N/A	N/A

*All cells are *n* (%)*.

**Figure 1:**
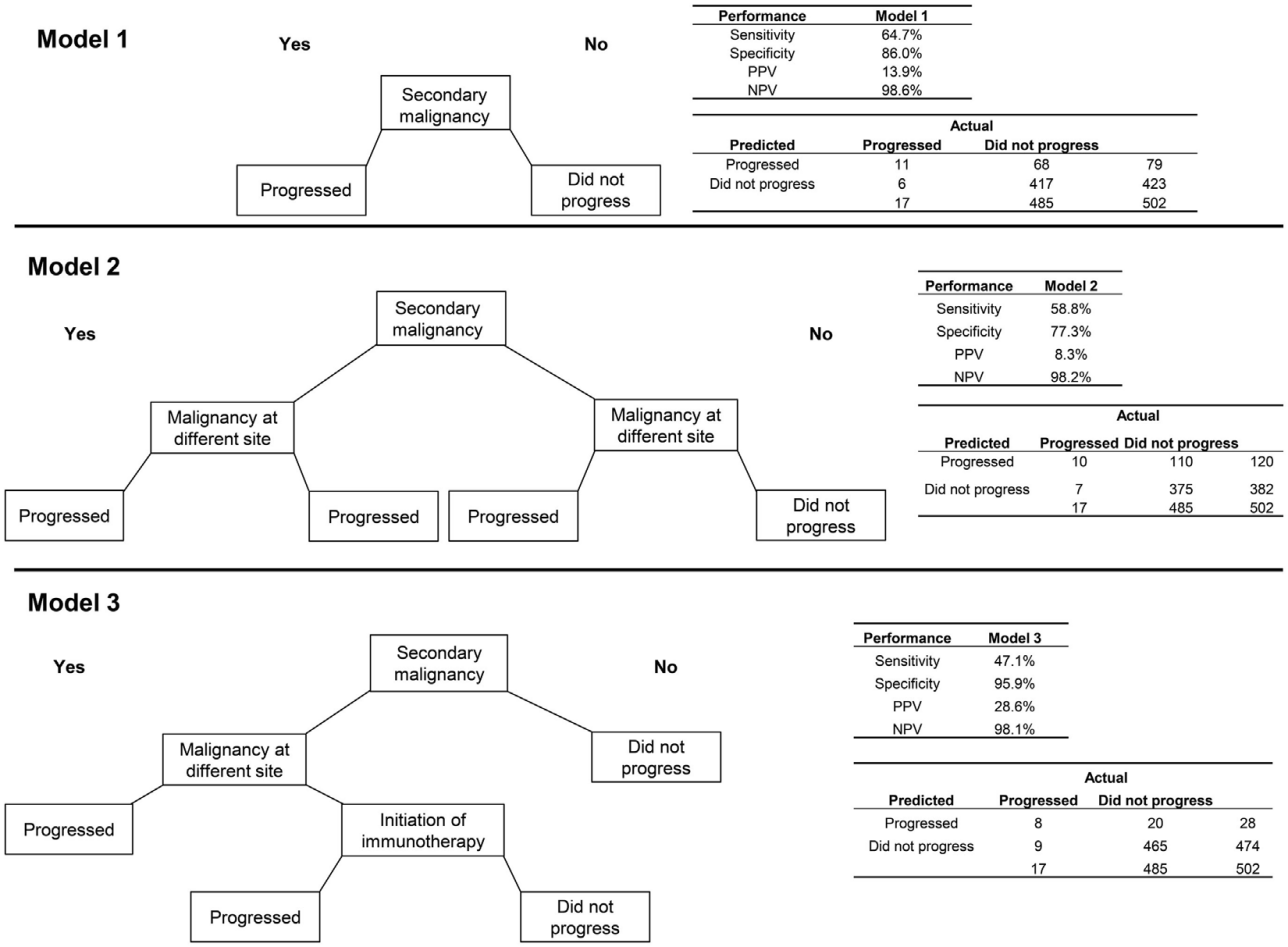
**Three algorithms for identifying progression to metastatic cancer among breast cancer patients**. In each decision tree, “yes” responses are to the left and “no” to the right. Model 1 is the presence of an ICD-9 code for secondary malignancy. Model 2 includes secondary malignancy or a code for malignancy at a different site than breast as indicators of progression. For Model 3, secondary malignancy diagnosis plus either a malignancy at another site or initiation of immunotherapy indicate progression.

First, an algorithm consisting of only the indicator for a secondary malignancy (i.e., the most appropriate coding of metastatic cancer using ICD-9 codes) was assessed (Figure 1). This algorithm had a sensitivity of 64.7% and specificity of 86.0%. Because of the very low incidence of progression to stage IV disease in breast cancer, the PPV was low, at 13.9%, with much higher NPV (98.6%).

Adding the variable for a second primary tumor at a different site from the breast to the secondary malignancy diagnosis, so that patients with either of these indicators were classified as having progressed, decreased the performance of the algorithm on all measures relative to the algorithm consisting of the secondary malignancy code alone. A more successful algorithm required the secondary malignancy diagnosis to be combined with either a malignancy at another site or initiation of immunotherapy. This last model obtained higher PPV (28.6%) and specificity (95.9%) but lower sensitivity (47.1%) than the model with secondary malignancy alone.

Sensitivity analyses delaying the start of the time window for qualifying radiology/pathology claims and subsequent elements obtained somewhat higher specificity and PPV than the 60-day results, but at the expense of some sensitivity. These analyses also required the exclusion of patients who progressed or were lost to follow-up within a year after the initial diagnosis.

#### Non-Small Cell Lung Cancer

Progression to stage IV cancer was more common in NSCLC than breast cancer, with 61 (25.8%) of patients having this outcome. Again, nearly all patients with NSCLC had a radiology or pathology claim at ≥60 days after the index date (Table 2). The differences between patients who progressed and those who did not were not as consistent as with the breast cancer patients, although many variables showed sizeable differences, including secondary malignancy diagnosis, malignancy at a different site, use of pain medication, initiation of radiation therapy, change in chemotherapy regimen, and initiation of a metastatic-specific therapy. The variable importance values obtained from RF supported the use of secondary malignancy diagnosis, malignancy at a different site, and change in chemotherapy regimen.

**Figure 2:**
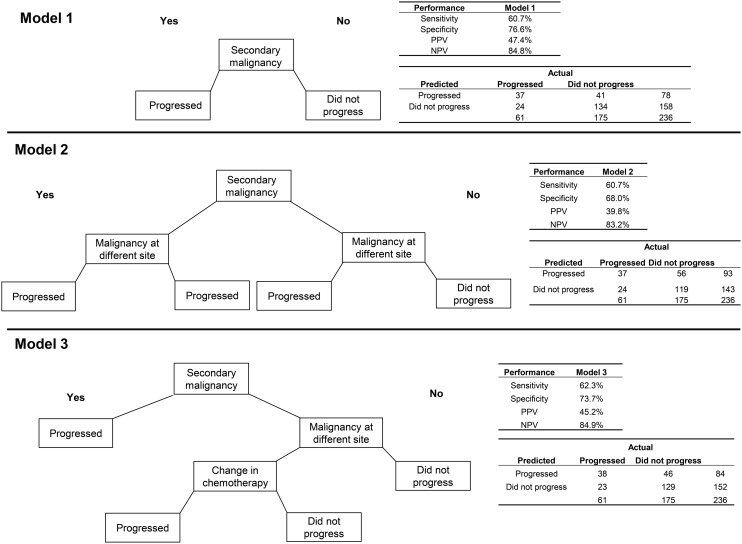
**Three algorithms for identifying progression to metastatic cancer among NSCLC patients**. Model 1 is the presence of an ICD-9 code for secondary malignancy. In Model 2, secondary malignancy or a code for malignancy at a different site than lung indicate progression. Model 3 includes either secondary malignancy diagnosis or a malignancy at a different site plus a change in chemotherapy as markers of progression.

The algorithm using only secondary malignancy performed better than the same model for breast cancer with respect to PPV (47.4%) but slightly worse on each of the other performance metrics (Figure 2). Adding malignancy at a different site identified no additional true positives and misclassified an additional 15 patients who did not progress, leading to poorer performance on all measures except sensitivity. A more complex model, which classified patients as having progressed if they had a secondary malignancy diagnosis or a malignancy at a different site plus a change in chemotherapy, performed about the same as the model with secondary malignancy alone. For lung cancer, the sensitivity analysis requiring longer delays prior to the earliest qualifying radiology/pathology claims led to consistently higher error rates, and so models using those other time windows were not explored.

#### Colorectal Cancer

Among patients with CRC, 33 (11.8%) progressed to metastatic cancer, all of whom had a radiology or pathology claim at least 60 days after initial diagnosis (Table 2). The secondary malignancy diagnosis was again prominent among those who progressed, with other variables appearing to discriminate including malignancy at a different site, biopsy or surgery of a different site, radiation therapy, change in chemotherapy regimen, and initiation of metastatic-specific therapy. The RF procedure found that secondary malignancy diagnosis and change in chemotherapy were the only two variables of high importance.

**Figure 3:**
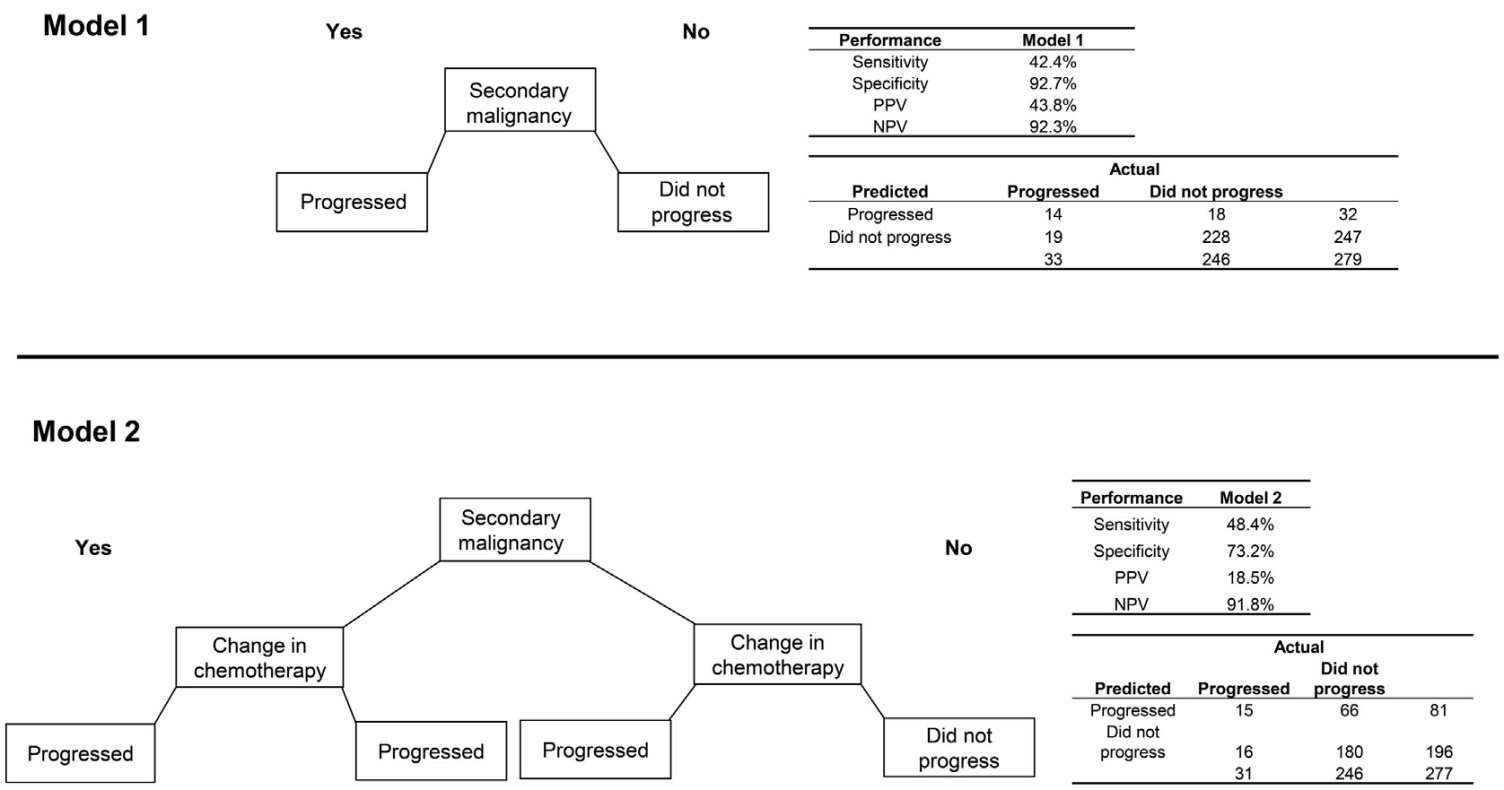
**Two algorithms for identifying progression to metastatic cancer among colorectal cancer patients**. Model 1 is the presence of an ICD-9 code for secondary malignancy. Model 2 classifies patients as progressed if they have either a secondary malignancy code or a change in chemotherapy regimen.

The model using only secondary malignancy diagnosis had good specificity (92.7%) and NPV (92.3%) but low sensitivity (42.4%) and PPV (43.8%; Figure 3). A model classifying patients with either a secondary malignancy or a change in chemotherapy had slightly better sensitivity but poorer performance on the other metrics. The sensitivity analysis requiring longer delays prior to the radiology/pathology claims had slightly lower error rates. Using a window of ≥120 days with the second model (secondary malignancy or change in chemotherapy), the PPV increased to 34.0% and specificity to 87.3%, with values for sensitivity (50.0%) and NPV (93.0%) that were similar to the 60-day model.

### Discussion

Progression in the stage of cancer may be an important early indicator of the effectiveness or lack of effectiveness of oncology therapy. Algorithms allowing identification of this outcome in routinely collected data would add significantly to our ability to conduct effectiveness and/or safety research in oncology. The present study utilized a hybrid clinical-empirical approach to develop algorithms for identifying progression to metastatic cancer in claims data. The performance of the algorithms was suboptimal, limiting their usefulness in future research, but the methods and findings may provide valuable guidance for further investigation into cancer progression.

In the ICD-9 coding scheme, metastatic cancer should be indicated by a secondary malignancy diagnosis code (197–199). Logically, the first occurrence of such a code should correspond to the date when progression to stage IV cancer was first identified. The present findings, however, demonstrate the serious imprecision of this approach. Taking a time window for first occurrence of a secondary malignancy code of 90 days before to 90 days after the actual date of progression, 65% or fewer progression events were successfully identified. At the same time, many patients who did not progress were incorrectly identified as having progressed.

Through the additional consideration of many variables deemed to have high clinical relevance to the identification of cancer progression, and using validation *via* a robust statistical method that is able to maximize the use of limited datasets, more complex algorithms were built. Diagnosis codes for a second primary tumor at a different site than the original malignancy were frequently seen in the claims data for patients who progressed. The RF procedure found a high variable importance for this indicator for two of the three tumor types (breast and NSCLC). Some clinical coders may be entering metastatic sites using the primary rather than secondary malignancy codes, although adding this code to the progression algorithms added too many false positives to be useful. Still, considering the relative rarity of multiple primary tumors of different sites (24), any patient with claims for more than one primary tumor type should at least be considered a possible case of metastatic cancer. Changes in antineoplastic treatment regimens can serve as additional flags for possible metastatic cancer status.

The RF procedure inherently uses cross-validation as it tests the accuracy of each tree by calculating the accuracy using the patients not used to build the tree. This allowed us to more efficiently use our small sample for both training and validation. In addition, the group sizes were pre-balanced prior to running each forest, which is a form of cross-validation. The initial analysis results consisted of the average performance over 100 such forests. Nevertheless, the number of patients who progressed, especially in the breast cancer cohort, was low enough to limit the usefulness of any algorithm developed in this study. In addition, the specificity and NPV of the algorithms is relatively uninformative, given the low proportion of patients who progressed; interpretation of the results should focus on the PPV and sensitivity.

Other limitations include the potential for missing data, in both the cancer registry and claims data sources. Diagnoses of stage IV cancer that occurred outside of the Geisinger Health System would be missing from the cancer registry and chart review. Missing claims data can lead to failure to identify patterns in claims that would more accurately distinguish between metastatic and earlier stage cancer. Treatment, diagnoses, and procedures that occurred but were not included in claims because they did not lead to billable medical care or were covered by dual insurance could not be identified as missing and were assumed not to have occurred. With the knowledge that claims data are imperfect, this study was performed to assess whether claims-based algorithms can be created to identify progression to metastatic cancer.

A minimum of 3 months of follow-up was required for patients to identify cancer progression to metastatic cancer. The results thus may not reflect the patterns of care for patients who died or disenrolled from the health plan shortly after initial diagnosis. The study population was derived from a single-state health plan and health system and may not be generalizable to the United States population. The reference standard used in this study was based on a manual chart review, which is an excellent source of information but still imperfect, especially considering the complexities of cancer staging and the potential for missing progression events that occurred. The overall rate of progression was lower than expected, which may have resulted from patients with advanced cancer seeking care from an oncology specialty center outside of the Geisinger system, or from progression events that were missed during the chart review.

It is possible that better-performing algorithms could have been developed using different machine learning techniques or by creating more empirically driven variables, identified through an exhaustive search of all claims for patients who progressed and those who did not. Still, an empirically based algorithm that lacks the clinical perspective offered in our present efforts may suffer from poor clinical validity as well as weighing too heavily the idiosyncrasies of the patient data included in the study sample.

## Conclusion

The results of this study suggest that identification of progression to distant metastatic cancer is not likely to be valid using claims data alone. There are limitations to each of the information sources that jeopardize the development of a consistently high-performing algorithm. Additional information, derived from medical records or cancer registries, is needed for accurate ascertainment of this outcome.

## Author Contributions

BN participated in the study concept and design, interpreted the data, and drafted the manuscript. JS, MD, ZK, JP, and HX participated in the study concept and design and interpreted the data. KM and KF extracted, managed, and analyzed the data. All authors provided critical revisions and approved the final manuscript.

## Conflict of Interest Statement

Beth L. Nordstrom, Jason C. Simeone, Karen G. Malley, and Kathy H. Fraeman are employees of Evidera, who received funding from Amgen for this study. Hairong Xu, Mark Durst, John H. Page, and Zandra Klippel are employed by and own equity in Amgen, Inc.

## Abbreviations

EHR, electronic health record; ICD-9, International Classification of Diseases, ninth revision; ICD-O-3, International Classification of Diseases for Oncology, 3rd edition; IQR, interquartile range; NPV, negative predictive value; NSCLC, non-small cell lung cancer; PPV, positive predictive value; RF, random forests; SEER, surveillance, epidemiology, and end results.
